# Low-dose splenic irradiation in symptomatic congestive splenomegaly: report of five cases with literature data

**DOI:** 10.1186/1748-717X-9-86

**Published:** 2014-03-27

**Authors:** Frank Bruns, Michael Bremer, Arne Dettmer, Stefan Janssen

**Affiliations:** 1 Department of Radiation Oncology, Hannover Medical School, 30625 Hannover, Germany; 2 Department of Gastroenterology, Hepatology and Endocrinology, Hannover Medical School, Hannover, Germany

**Keywords:** Splenic irradiation, Congestive splenomegaly, Symptomatic, Radiotherapy, Benign disease

## Abstract

**Background:**

To show effectiveness of low-dose splenic irradiation in symptomatic congestive splenomegaly.

**Methods:**

Five patients were referred to our department for symptomatic congestive splenomegaly within three years. Primary diseases were autoimmune hepatitis with liver cirrhosis (n = 2), cystic fibrosis (n = 1), granulomatous liver disease (n = 1) and Werlhof disease with liver cirrhosis (n = 1). Mean age was 54 years (range: 36–67). Patients received splenic irradiation with a total dose of 3 Gy (single dose: 0.5 Gy). One patient was re-irradiated after long-term failure with the same treatment schedule.

**Results:**

In four patients long term relief of splenic pain could be observed during the follow-up time of median 20 (range: 2–36) months. Four patients showed haematological response after irradiation with an increase of erythrocytes, leucocytes and/or platelets. A slightly decrease in spleen size was found in two patients.

**Conclusions:**

Low-dose splenic irradiation in symptomatic congestive splenomegaly is feasible and perhaps as effective as in lympho-and myeloproliferative malignancies regarding pain relief and haematological response.

## Background

Splenic irradiation has been widely used in palliative treatment of symptomatic splenomegaly in patients with lymphoid and myeloid malignancies as reported currently by Kriz et al. in a large cohort of patients using different fractionation regimens [1]. Apart from lymphoid and myeloid malignancies hypersplenism–characterized by painful splenomegaly and cytopenia–can also occur as a secondary phenomenon in a variety of benign disorders. Beside liver transplantation as solely curative treatment of the primary liver disease, splenectomy and minimal invasive procedures such as splenic embolisation are well-tried treatment options for patients with symptomatic congestive splenomegaly, e.g. secondary due to portal hypertension from liver cirrhosis. However, these treatment options carry a high risk of possible complications [2]. Splenic irradiation constitutes an alternative non-invasive treatment option. Recently, Weinmann et al. analyzed splenic irradiation in autoimmune disorders like autoimmune thrombocytopenia or autoimmune hemolytic anemia with promising results [3]. Splenic irradiation for symptomatic congestive splenomegaly is unusually and there are only two reports on this subject in the Anglophone literature. This report of five patients aims to confirm the published data in particular regarding the potential of symptom control.

## Methods

In the recent four years, five patients (female: 2, male: 3) with symptomatic congestive splenomegaly received splenic irradiation as an individual treatment approach. Patients had contraindications for alternative treatment options or refused interventional approaches. Primary diseases were autoimmune hepatitis with consecutive liver cirrhosis (n = 2), cystic fibrosis (n = 1), granulomatous liver disease (n = 1) and M. Werlhof with liver cirrhosis (n = 1). Mean age was 54 years (range: 36–67). All patients and treatment parameters are summarized in Table 1.

**Table 1:** Patients and treatment characteristics

**Patient number**	**1**	**2**	**3/3a**	**4**	**5**
Sex (male/female)	m	m	f	f	m
Age (years)	56	36	49	61	67
Primary disease	Autoimmune hepatitis	Cystic fibrosis	Autoimmune hepatitis	Granulomatous liver disease	M. Werlhof with liver cirrhosis
Single/total dose	0.5/3.0 Gy	0.5/3.0 Gy	0.5/3.0 Gy Second course (3a): 0.5/3.0 Gy	0.5/3.0 Gy	0.5/3.0 Gy
Follow-up (months)	21	35	24 (36) and (3a) 12	7	2

Radiation was delivered after three dimensional (3D) Computertomography-based treatment planning using 6-or 10 MV linac photons. Total dose given was 3.0 Gy with single fractions of 0.5 Gy two or three times a week. Patient 3 had a second course of splenic irradiation after long-term failure (progressive pain, 2 years after radiotherapy) with the same treatment schedule.

Beside pain evaluation (four-point scale: no pain-mild pain-moderate pain-severe pain) size measurement of the spleen was performed by sonography. Blood counts were monitored before each fraction and during follow-up visits.

### Consent

Informed consent was obtained from the patient for the publication of this report.

## Results

Median follow-up was 20 months (range: 2–36). The results are summarized in Table 2. In all patients with precedent splenic pain long term pain relief could be observed (decrease from severe pain to no pain in patient 2; from mild pain to no pain in patient 1 and 3 [second serie]; and from moderate pain to mild pain in patient 5); in two patients with painless abdominal feeling of pressure no improvement arose. A slight decrease in splenic size of about 10% was seen in 2 patients (patient 1: length: 24 cm to 22 cm; patient 2: length: 21.6 cm to 20.0 cm); in two further patients the splenic size was stable after a short temporary decrease. For two patients (including patient 3 after second irradiation) no follow-up sonography was available to date. Hematological response after irradiation with an increase of erythrocytes, leucocytes and/or platelets could be observed in four patients (see Table 2). Patient 2 had a decrease of blood cells immediately after irradiation but hematological parameters remained stable during long term follow-up; no hematological effect was observed in patient 3 after second irradiation. Neither acute side effects of irradiation like nausea nor any late sequela has occurred so far.

**Table 2:** Treatment results

**Patient number**	**1**	**2**	**3**	**3a**	**4**	**5**
Relief of splenic pain	+	+	*	+	*	+
Haematological response	+	–	+	–	+	+
Decrease of spleen size	+	+	–	n.a.	–	n.a.
WBC before/after RT	1.9/2.1	5.0/3.9	1.6/2.5	3.7/2.4	3.3/4.1	2.5/2.6
	Δ +11%	Δ –22%	Δ +57%	Δ –35%	Δ +25%	Δ +4%
Hb before/after RT	11.7/14.1	12.0/12.7	9.1/12.1	12.7/11.2	12.5/13.6	11.4/11.2
	Δ +21%	Δ +6%	Δ +33%	Δ –11%	Δ +9%	Δ –1%
Plts before/after RT	36/44	24/13	24/30	29/29	72/91	37/52
	Δ +23%	Δ –45%	Δ +25%	Δ ±0%	Δ +27%	Δ +38%

WBC: white blood cells (10^3^/μl), Hb: hemoglobin (g/dl), Plts: platelets (10^3^/μl), *painless feeling of abdominal pressure, −: no effect, n.a.: not available.

## Discussion

Splenomegaly is a frequent finding in patients with liver disease due to venous congestion as a consequence of portal hypertension; hypersplenism is characterized by congestive splenomegaly and secondary a reduction of erythrocytes, leucocytes and/or platelets due to splenic pooling/sequestion [2]. In patients with poor liver function a splenectomy is often not possible. Alternatively, mimimal invasive procedures such as partial splenic embolisation and transjugular intrahepatic portosystemic shunt (TIPS) implantation are used to reduce portal venous pressure [2,4,5]. Radiofrequency ablation (RFA) and microwave (MW) ablation of splenic parenchyma – as less radical techniques–are also applicable in symptomatic congestive splenomegaly [6,7]. None of these procedures provide convincing results and are all associated with the potential of major complications including splenic infarction, abscess formation, pain, bleeding and sepsis.

Splenic irradiation is a well-established palliative treatment option in patients with lympho-and myeloproliferative malignancies presenting with symptomatic splenomegaly [1,8]. In addition, radiotherapy is known to be effective in a large variety of degenerative and hyperproliferative benign conditions [9]. In terms of hypersplenism due to splenic congestion the value of radiotherapy is limited to two case series as illustrated below [10,11].

In all patients with precedent splenic pain treated with radiotherapy we observed a long-term pain relief. In addition we found an improvement of hematological parameters, particularly of platelets in four patients and a slightly decrease of spleen size of about 10% in two patients.

Until now, only two case series are reported in the Anglophone literature, which analyse 5 and 8 patients respectively treated with splenic irradiation for symptomatic congestive splenomegaly [10,11]. Both achieved outcomes similar to ours in terms of pain relief (Kenawi: 8 of 8 patients [100%]; Liu: 2 of 2 [100% of the patients with precedent pain]; 3 patients had precedent painless splenomegaly) and increase of platelets (Kenawi: 3 of 8 patients; Liu: 5 of 5 patients) after splenic irradiation. The mean follow-up of 4 and 19 months respectively was quite short compared to 20 months in our analysis. The applied total dose was significantly higher with 12 Gy in 8 fractions (single doses of 1.5 Gy, five times a week) in the serie of Liu et al. [11], while Kenawi et al. [10] applied different total doses from 3.0 to 23.0 Gy (mean total dose approximately 12 Gy; single doses ranged from 0.5 to 1.5 Gy). Moreover, in all reported patients there was no correlation between clinical response and change in spleen size observed.

Osorio et al. [12] reported another case of painful splenomegaly due to Eisenmenger’s syndrome. Their patient was treated with splenic irradiation with a very high total dose of 40 Gy and daily single doses of 2.5 Gy. The patient achieved good pain relief and marked reduction of spleen size but he died 4.5 months after radiotherapy due to complications of acute cholecystitis. However, very high-dose splenic irradiation as performed in this case is debatable and seems inappropriate to achieve symptom control in such a palliative setting.

For splenic irradiation in lympho-and myeloproliferative malignancies a large variation of different schedules is used with single doses from 0.1 to 2 Gy and total doses of 0.3–16 Gy and no consensus recommendations exist concerning total dose and fractionation regimes until now [1,13]. In our series we showed that a significant lower dose of 3.0 Gy is sufficient to achieve suitable pain relief and hematological response compared to higher doses (12 Gy and more) reported by Kenawi and Liu et al. In addition, prescription of lower doses preserves the potential of re-irradiation in cases of treatment failure in this palliative setting, as it was seen in patient 3.

The exact mechanism how splenic irradiation exerts its effects in splenomegaly is poorly understood. While splenic irradiation is usually considered as a local treatment only with direct effects on the spleen, it also induces systemic effects, which includes problematic symptoms and life-threatening pancytopenia; mechanisms believed to contribute to these effects include direct radiation-induced cell death, immune modulation via selective reduction in lymphocyte subsets, and cytokine induction [13,14].

So far, in accordance with the literature data we observed in our case series no clinically relevant early or late toxicity. Particularly, no hematologic toxicities > CTC II° occurred.

## Conclusions

As shown for lympho-and myeloproliferative malignancies before, low-dose splenic irradiation seems to be effective in treating symptomatic congestive splenomegaly for patients with contraindications for splenectomy and embolisation or refusal of interventional treatment. This safe and well-tolerated treatment warrants further study.

## Competing interests

The authors declare that they have no competing interests.

## Authors’ contributions

FB, MB and SJ treated patients in this study. SJ and AD have made substantial contributions to acquisition and interpretation of data. FB and SJ drafted the manuscript. All authors read and approved the final manuscript.
